# Assessment of Blood Flow Velocity in Retinal Vasculitis Using the Retinal Function Imager—A Pilot Study

**DOI:** 10.3390/jcm13133975

**Published:** 2024-07-08

**Authors:** Nicole Stuebiger, Wen-Hsiang Lee, Johannes Birtel, Vasyl Druchkiv, Janet L. Davis, Delia Cabrera DeBuc

**Affiliations:** 1 Department of Ophthalmology, University Medical Center Hamburg-Eppendorf, 20246 Hamburg, Germany; 2 Bascom Palmer Eye Institute, University of Miami, Miami, FL 33136, USA; 3 Department of Ophthalmology, University of Bonn, 53127 Bonn, Germany

**Keywords:** posterior uveitis, birdshot chorioretinopathy, retinal vasculitis, retinal function imager, retinal blood flow velocity, non-invasive capillary perfusion maps

## Abstract

**Background:** This pilot study aimed to evaluate the Retinal Function Imager (RFI) for visualizing retinal vasculature and assessment of blood flow characteristics in patients with retinal vasculitis. The RFI is a non-invasive imaging device measuring the blood flow velocity (BFV) in secondary and tertiary retinal vessels using hemoglobin as an intrinsic motion-contrast agent. **Methods:** To test the feasibility of the RFI for patients with retinal vasculitis, capillary perfusion maps (nCPMs) were generated from 15 eyes of eight patients (five females; mean age: 49 ± 12 years) with a mean uveitis duration of 74 ± 85 months. Five of these patients had birdshot chorioretinopathy, and three had primarily non-occlusive venous retinal vasculitis of unknown origin. To reflect that the BFV may be more reduced in patients with prolonged disease, patients were classified into a short-term (uveitis duration: 8–15 months) and a long-term uveitis group (uveitis duration: 60–264 months). Data were compared with healthy controls (16 eyes of 11 patients; mean age 45 ± 12 years; 8 females). **Results:** The mean BFV in the controls was 3.79 ± 0.50 mm/s in the retinal arteries and 2.35 ± 0.44 mm/s in the retinal veins, which was significantly higher compared to the retinal vasculitis group. Patients revealed an arterial BFV of 2.75 ± 0.74 mm/s (*p* < 0.001) and a venous BFV of 1.75 ± 0.51 mm/s (*p* = 0.016). In the short-term group, a trend towards a decreased venular and arteriolar BFV was seen, while a significant reduction was observed in the long-term group. The patients’ microvasculature anatomy revealed by the nCPMs appeared unevenly distributed and a lower number of blood vessels were seen, along with a lower degree of complexity of their branching patterns, when compared with controls. **Conclusions:** This study demonstrated a reduction in venular and arteriolar BFVs in patients with retinal vasculitis. BFV alterations were already observed in early disease stages and became more pronounced in progressed disease. Additionally, we showed that retinal microvasculature changes may be observed by nCPMs. Retinal imaging with the RFI may serve as a diagnostic and quantifying tool in retinal vasculitis.

## 1. Introduction

Retinal vasculitis (RV) is a rare intraocular inflammatory condition often leading to vision impairment or blindness. Its pathophysiology includes the disruption of tight endothelial junctions which may cause fibrosis, narrowing, and/or occlusion of the retinal vasculature, resulting in capillary non-perfusion, retinal ischemia, and development of macular edema [1,2,3].

RV is commonly associated with systemic diseases such as Behçet’s syndrome, lupus erythematosus, multiple sclerosis, or sarcoidosis, but may also occur as an isolated entity—such as idiopathically or as part of an ocular syndrome [1,3]. One of the so-called “ocular syndromes”, which, by definition, does not have systemic manifestation is birdshot chorioretinopathy (BSCR) [4]. BSCR belongs to the white dot syndromes, where chorioretinitis with yellowish punctate infiltrates occurs in addition to retinal vasculitis, which is characterized as primarily non-occlusive with venule predominance, but also occlusive vascular changes are observed [3,4,5,6].

The routine approach to investigate retinal vasculitis is fluorescein angiography (FAG); however, this invasive technique has drawbacks and primarily provides insights only into the retinal microvascular function [7,8,9]. Additional imaging modalities include optical coherence tomography–angiography (OCT-A) [7,10,11,12,13], doppler OCT [14,15,16,17,18,19], ultrasound color doppler [20,21], and approaches such as the Heidelberg retina flowmeter [22], laser doppler velocimetry [23,24], Canon Laser blood flowmeter [25], and adaptive optics scanning laser ophthalmoscopy (AOSLO) [26,27]. However, all these modalities include a high variability between measurements, extended examination times, measurements of only small areas of interest, or velocity only in vessels with larger diameters, and a lack of information on the flow velocity [23,24]. 

Some of these limitations may be overcome by the Retinal Function Imager (RFI), which non-invasively evaluates and quantifies retinal hemodynamics. It measures blood flow velocities (BFVs) in secondary and tertiary branches of the retinal vessels. A unique feature of the RFI is the possibility to study flow velocity changes in a large range of arterioles and venules simultaneously, with a field view of 35° [8,9,28,29,30,31,32,33,34,35,36]. Furthermore, it has a high reproducibility [31,37] and provides capillary perfusion maps (nCPMs) [8,9,28,29,30,31,32,33,34,35,36]. Unlike depth-encoded images from OCT-A, the RFI cannot differentiate the depth between different vascular networks due to the nature of en-face imaging [9,38].

So far, the RFI has been used in various diseases such as diabetic retinopathy [36,38,39,40,41], glaucoma [42,43], non-arteritic anterior ischemic optic neuropathy [44], age-related macular degeneration [45,46,47,48], central serous chorioretinopathy [49], idiopathic juxtafoveal telangiectasia [50], and in anterior uveitis [51]. 

As the RFI may hold value in patients with retinal vasculitis, this pilot study evaluated the BFV imaging and the capabilities of this imaging device in patients with retinal vasculitis, in comparison to a healthy control group.

## 2. Patients and Methods

The study was approved by the Institutional Review Board of the University of Miami, Florida, USA (IRB protocol no. 20130296) and was conducted in accordance with the Declaration of Helsinki. All participants provided written informed consent. 

### 2.1. Patients

All subjects had retinal vasculitis with or without posterior uveitis and underwent comprehensive ophthalmic examination, including medical history, assessment of best-corrected visual acuity (BCVA), measurement of intraocular pressure (IOP), indirect ophthalmoscopy, OCT imaging and fluorescein- and/or indocyanine green angiography. The blood pressure was measured in all subjects and was always within the normal range.

The uveitis workup included total blood count, c-reactive protein (CRP), erythrocyte sedimentation rate (ESR), rheumatoid factor, antinuclear antibodies, anti-neutrophil cytoplasmatic antibodies, anti-double strained DNA-antibodies (dsDNA-Ab), angiotensin-converting enzyme, soluble-interleukin-2-receptor (s-IL-2R), Cytomegalovirus (serology), Herpes simplex (serology), Borreliosis (serology), Syphilis (serology), Tuberculosis (Quantiferon^®^ TB Gold Test) and Toxoplasmosis (ELISA). 

As the BFV may depend on the uveitis duration and on the presence of intraretinal fluid [51], patients were divided into a short term (≤15 months; n = 6 eyes) and a long term (≥60 months; n = 9 eyes) group. As only one patient showed macular edema (Table 1, pat. #4), we did not further subdivide the patients according to this uveitic complication.

The control group (11 healthy volunteers, 16 eyes; mean age 45 ± 12 years; 8 females; mean visual acuity 0 ± 0.01 logMAR) was age and gender matched; none had known ocular or systemic disease, and their blood pressure was in all within normal range.

### 2.2. Retinal Function Imager

The Retinal Function Imager (RFI-3005, Optical Imaging, Rehovot, Israel) has been described before [9,28,29,30,31,36,38]. In short, it consists of a fundus camera, a stroboscopic lamp system, and a camera. For the BFV mode, a green (=red-free) interference filter with transmission centered at 548 nm and a bandwidth of 17 nm is used. By comparing eight frames (short movies) under a fast stroboscopic illumination at a wavelength strongly absorbed by hemoglobin in erythrocytes, the erythrocytes’ motion is measured within an interval of 0.14 s (17.5 ms between flashes). The size of this offset, suitably calibrated and multiplied by the frame rate, gives BFV in millimeters per second (mm/s). Additionally, non-invasive capillary perfusion maps (nCPMs) are generated by the digital enhancement of the alignment of those static frames. The capillary perfusion maps provided high-resolution images of the retinal microvasculature, showing fine capillary changes in the macular and perimacular region [33,34].

### 2.3. Imaging and Image Analysis

**Figure 1:**
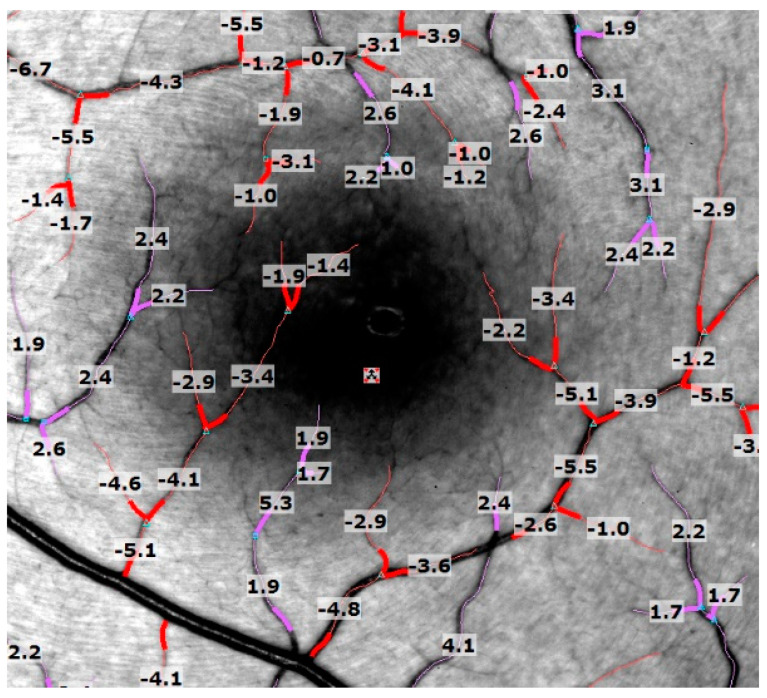
Fundus and blood velocity map in a healthy subject. Velocities are measured in veins (violet; positive values) and in arteries (red; negative values) and presented in millimeters per second average ± standard deviation (SD).

All the subjects underwent RFI imaging in a separate session by a single experienced photographer. Before imaging, their blood pressure was measured. The RFI recorded the heart rate directly by attaching a probe to the subject’s finger, allowing for synchronized image acquisition to the patient’s pulse patterns. After pupillary dilation, five to seven serial RFI images were acquired, centered on the fovea with a field view of 35°. After 15–20 min of imaging, a single observer assessed the quality of the images, low-quality images were not included in this study. Images with visible blood flow in the “ratio mode” were used for analysis; multiple segments, around 12–20 per eye, of secondary and tertiary branches of the main vessels (arteries and veins) were selected. To avoid blood vessel crossings, we choose a blood vessel length of ≤100 µm. Peripheral segments, which were mainly unfocused, were excluded. The built-in software measured each segment’s average velocity and relative standard coefficient of variance, calculated as standard deviation over the mean. A negative value indicates blood flow away from the heart, whereas a positive value suggests blood flow toward the heart (Figure 1). Vessel segments with a relative standard deviation of more than 0.45 were automatically highlighted, and consecutively deleted, by the built-in software as they were considered unreliable.

### 2.4. Statistical Analysis

When analyzing the individual segment velocities, a logarithmic transformation to transform the velocity data into a normal distribution was used. Given the hierarchical data structure (eyes nested within patients), a mixed regression model with a random patient effect and maximum likelihood estimation method was fitted separately for the arteries and veins from the retina [52]. In this way, standard errors could be adjusted and eventual correlation within the patients could be modeled. The differences between the marginal means were then tested using a covariance structure from the model above and degrees of freedom adjusted with the containment method [53]. The t-statistic was used to derive *p* values, which were then adjusted with Tukey’s method for multiple comparisons, when we compared three groups (long-term, short-term and control) against each other. All analyses were performed using R Core Team 2021 [54]. The significance was set at *p* < 0.05.

## 3. Results

**Figure 2:**
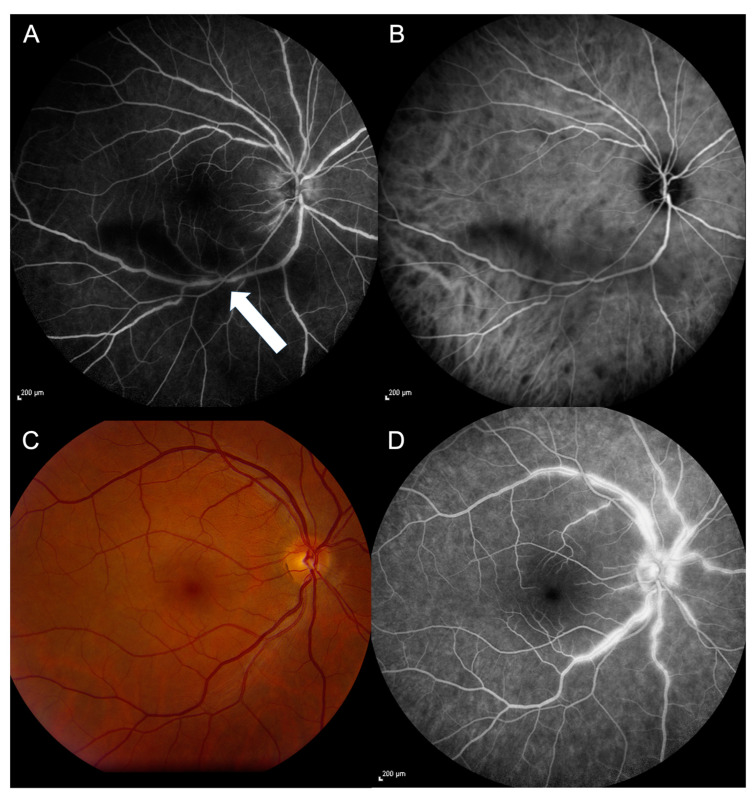
Fluoresceine- (**A**,**D**) and indocyanine-green angiography (**B**) of a patient (OD, #7) with birdshot chorioretinopathy. (**A**) shows slight leakage of the inflamed venous vessels (white arrow) in the FA. The characteristic dark dots (inflammatory changes in the choriocapillaris) are visible in indocyanine-green angiography (**B**). (**C**) shows the fundus of patient (OD, #8) with non-occlusive retinal vasculitis of unknown origin; (**D**) the characteristic dye leakage through the disrupted tight endothelial junctions of the inflamed retinal veins when performing fluorescein angiography.

This study initially included 16 eyes of eight patients with retinal vasculitis (mean age 49 ± 12 years; five females); one eye had to be excluded due to severe retinal atrophy (#4, OD) leading to a cohort of 15 eyes with a median visual acuity of 0.1 ± 0.4 logMAR. Five patients (ten eyes) had HLA-A29 positive birdshot chorioretinopathy (Figure 2A,B), while three (five eyes) had primarily venous retinal vasculitis of unknown origin (Figure 2C,D). The mean duration of uveitis was 79 ± 85 months (range 8–264 months); six eyes (three patients, mean age 44 ± 5.0 years, two females) had short term (≤15 months) uveitis with a mean duration of 10 ± 3 months. The median visual acuity in the long-term group was 0.1 ± 0.37, and 0.05 ± 0.15 logMAR in the short-term group.

Despite systemic and/or topical therapy, 10 eyes revealed chronic persistent retinal inflammation on fluorescein angiography. Five eyes showed no vascular leakage. One patient (# 4) had bilateral cystoid macular edema (CME), confirmed by OCT imaging. Current systemic therapy included mycophenolate mofetil in two BSCR patients, where one BSCR patient received adalimumab. Five eyes (four patients) received intravitreal corticosteroid implants: four eyes with fluocinolone acetonide implant 0.59 µg, and one with a dexamethasone implant. Detailed demographic and clinical information of the study patients is provided in Table 1.

**Table 1:** Clinical characteristics of study subjects.

Pat. no.	Dx	HLA-A29	Uveitis Duration since Onset (m)	Age (y)	Sex	Affected Eye(s)	Course of Uveitis	FAG Findings	Uveitis Complications	Uveitis Tx (Always Months before Imaging)	Past Surgical History (Always Months before Imaging)	Concomitant Systemic Disease	BCVA (logMAR) OD/OS
1	BSCR	pos.	264	68	Female	OU	pers.	OU: 2;4	OU: retinal atrophy	Fa implant 0.59 mg:	Ahmed-Valve: OD 65 m	Migraine, HTN, Hypercholesterinemia	0.1/0.1
									OU: sec. glaucoma	OD: 2× (last 36 m)	CE: OD (74 m)		
									OU: CME in the past	OS: 3× (last 18 m)	CE: OS (126 m)		
2	BSCR	pos.	108	48	Female	OU	pers.	OU: 0	None	MMF 3 g/d (start: 17 m)	none	none	0.1/0.1
3	RVUO	neg.	108	44	Male	OU	pers.	OU: 1;4	OD: sec. glaucoma OD: sec. cataract	Fa implant 0.59 mg: OD: (78 m) Peg-IFN alpha sc (stop 57 m)	Removal of Fa implant (steroid response): OD (67 m)	none	0.2/0.1
4	BSCR	pos.	61	33	Female	OD > OS	pers.	OU: 3;4	OD > >OS: ret. atrophy OD: sec. cataract OU: CME	Dm implant: OD (16 m) TA ivi: OD (36 m) MMF: 1 g/d (start 38 m) IFX: 5 mg/kg BW iv, (stop 1 m)	CE: OD (18 m)	none	0.7/1.0
5	BSCR	pos.	60	67	Male	OU	pers.	OU: 0;4	OU: sec. cataract	Fa implant 0.59 mg: OU (8 m) MMF 2g/d (stop 14 m)	CE: OU (180 m)	none	0.6/0.1
6	RVUO	neg.	15	46	Female	OD >> OS	pers.	OU: 1;4	OD: sec. cataract OU: sec. glaucoma	ADA 40 mg sc/second week (start 2 m) IFX 5 mg/kg BW iv (7 times, stop 2 m) MMF 2 g/d (stop 3 m)	CE: OD (10 m) ppV(ret. detachment): OD (14 m)	Crohn’s disease, Hashimoto Thyreoiditis	1.0/0
7	BSCR	pos.	8	48	Female	OD > OS	pers.	OD > OS: 1	none	none	none	Chronic Fatique Syndrom	0.4/0
8	RVUO	neg.	8	39	Male	OS >> OD	pers.	OU:1 OS:4	none	none	none	none	0/0.1

Dx = diagnosis, BSCR = Birdshot Chorioretinopathy, RVUO = retinal vasculitis of unknown origin, m = months, y = years, Tx = treatment, pos. = positive, neg. = negative, BCVA = best corrected visual acuity; logMAR = Logarithm of the Minimum Angle of Resolution, RFI = Retinal Function Imager, OD = Oculus dexter, OS = Oculus sinister, OU = both eyes, pers. = persistent, sec. = secondary, ret. = retinal, CME = cystoid macular edema, MMF = mycophenolate mofetil, Ta = Triamcinolone acetonide, Fa = Fluocinolone acetonide, Dm = Dexamethasone, ADA = adalimumab, IFX = infliximab, Peg-IFN = pegylated interferon alpha, mg = milligram, g = gram, d = die, iv = intravenously, ivi = intravitreally, BW = bodyweight, CE = cataract extraction, IOL = intraocular lens, ppV = Pars Plana Vitrectomy, ret. = retinal, HTN = hypertonus, FAG = Fluoresceine Angiography: 0 = no vascular leakage/no vessel occlusion, 1 = vascular leakage, 2 = vessel occlusion, 3 = vascular leakage and vessel occlusion, 4 = quenching.

### 3.1. Retinal Blood Flow Velocities

**Figure 3:**
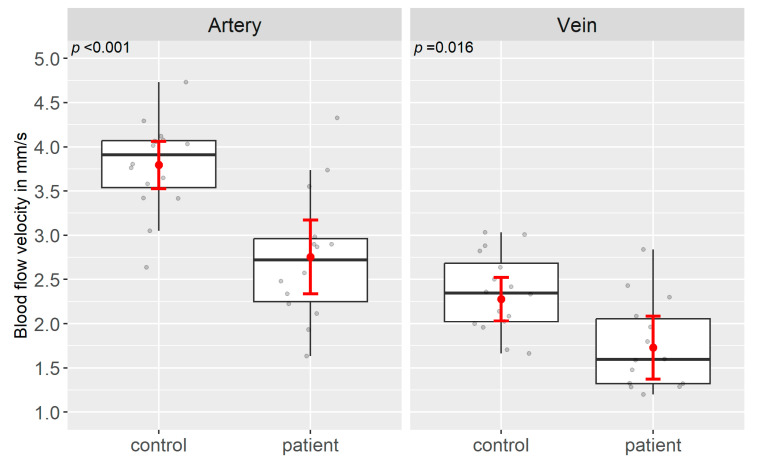
Box–Whisker Plot of the distributional characteristics of the BFV (in mm/s) in the patient and the control group (data see Table 2) with the mean BFV ± 95% confidence interval (red), the median of the BFV and the upper and lower quartiles (25% and 75% quartiles), the corresponding whiskers (black), and the individual patient eyes (grey). Comparing the patients arterial and venous BFVs to the control group, both are significantly lower in the patient group.

The mean BFV in patients in both the arteriolar (2.75 ± 0.74 mm/s), and venular segments (1.75 ± 0.51 mm/s) were significantly lower compared to controls (arteriolar: 3.79 ± 0.50 mm/s, *p* < 0.001; venular: 2.35 ± 0.44 mm/s, *p* = 0.016) (Table 2, Figure 3).

**Figure 4:**
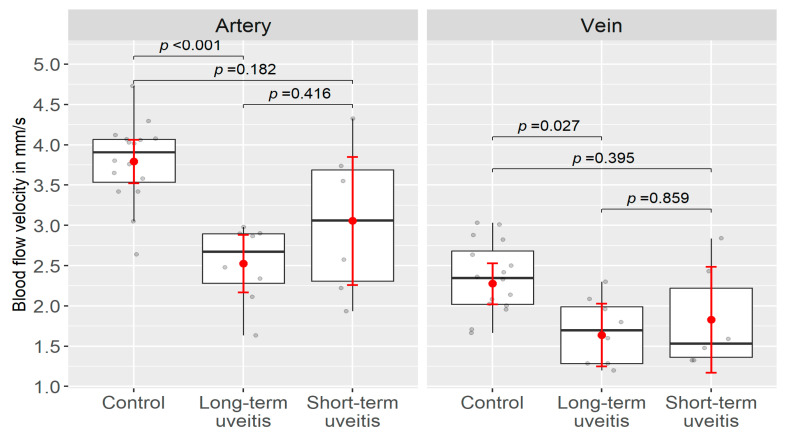
Box–Whisker Plot of the distributional characteristics of the BFV (in mm/s) in the short- term patient group, in the long-term patient group, and in the control group (data see Table 2) with the mean BFV ± 95% confidence interval (red), the median of the BFV and the upper and lower quartiles (25% and 75% quartiles), the corresponding whiskers (black), and the individual patient eyes (grey). Compared to controls no significant reduction in the blood flow velocity was seen in patients with short-term uveitis, while a significant decrease in the arteriolar and of the venular blood flow velocity was observed in patients with long-term uveitis.

Compared to the controls, patients with short- and long-term uveitis showed a lower BFV in the retinal arteries and veins, respectively. While no significant reduction in the BFV was seen in patients with short-term uveitis compared to controls (arterioles: *p* = 0.182; venules: *p* = 0.395), a significant decrease in the arteriolar (*p* < 0.001) and venular BFVs (*p* < 0.027) was observed in patients with long-term uveitis. Comparing these vasculitis subgroups, a trend towards lower BFV were observed in patients with long-term uveitis, both in the arteriolar BFV (*p* = 0.416) and in the venular BFV (*p* = 0.859) (Table 2, Figure 4).

### 3.2. Capillary Perfusion Maps/Blood Flow Velocity Maps

**Figure 5:**
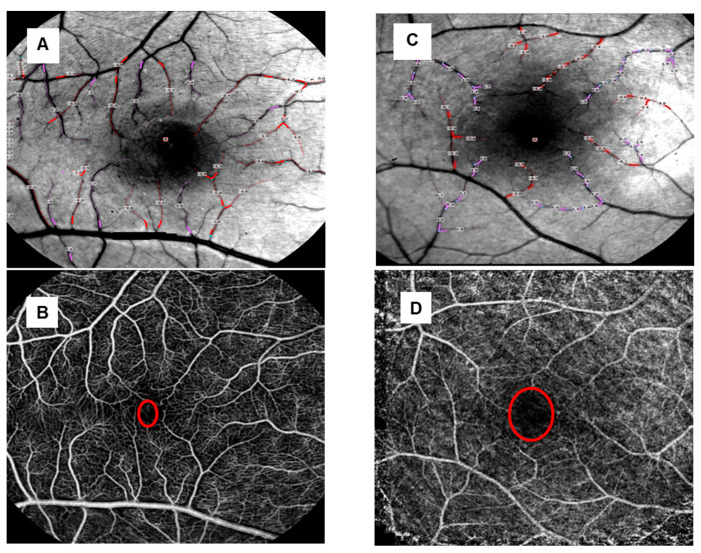
(**A**–**D**) Blood flow velocity maps and non-invasive capillary perfusion maps. (**A**) shows the BFV map of a healthy volunteer (OD), (**B**) the nCPM of the same eye; the red circle demonstrates the foveolar avascular zone (FAZ). (**C**) presents the BFV map of a BSCR patient (OD, pat.#7) and (**D**) the nCPM of the same eye. The FAZ (red circle) in (**D**) shows a larger diameter when compared to the FAZ of the healthy volunteer in (**B**).

The blood flow velocity maps demonstrated fewer arterioles and venules in the retinal vasculitis group compared to healthy controls (Figure 5A,C). Although the patients’ microvasculature anatomy revealed by the nCPMs appeared unevenly distributed, a lower number of blood vessels and a lower degree of complexity of their branching patterns were observed compared to normal healthy eyes (Figure 5B,D). Additionally, a larger foveolar avascular zone (FAZ) was visible in the patient’s eyes (Figure 5D). When comparing the short-term uveitis group and the long-term uveitis group, the reduction in microvasculature was more pronounced in the eyes of patients with a longer uveitis duration.

## 4. Discussion

The Retinal Function Imager is a non-invasive imaging system that is capable of quantifying retinal blood flow velocities in secondary and tertiary branches of arterial and venous vessels of the retina. Here, the RFI was used to evaluate BFV in patients with retinal vasculitis and demonstrated a reduction in venular and arteriolar BFVs compared to the controls. This BFV alteration may be attributed to inflammation-induced swelling, narrowing, quenching, and breaking down of retinal vessels. It was already observed in the early stages of vasculitis, while it grew more pronounced in progressed disease. Thus, imaging the retinal vasculature with the RFI may offer a diagnostic and quantifying tool for retinal vasculitis patients and a potential imaging modality for assessing the disease course and related treatment approaches.

Previously, Feng et al. [51] studied RFI-generated retinal BFVs from patients with anterior uveitis and showed that patients had decreased venous and arteriolar BFVs. Anterior uveitis patients with CME had an even higher reduction in the venous BFV and a reduced arterial BFV compared to controls. The authors interpreted such a reduction as an effect of inflammation on retinal vessels. The pronounced reduction in BFV in eyes with CME was thus associated with more severe or prolonged inflammatory processes which commonly appear later during the disease course [51].

To reflect that the BFVs may be more reduced in patients with extended disease, we classified our patients into two groups, a short-term (uveitis duration: 8–15 months) and long-term uveitis group (uveitis duration: 60–264 months). In the short-term group, a trend of decreased venular and arteriolar BFVs was seen, while a significant reduction was observed in the long-term group. This may support Feng et al. [51] in that severe retinal vessel alterations occur in more prolonged intraocular inflammation.

Additionally, when comparing the long-term to the short-term group venous BFV reduction was quite similar (1.7 ± 0.6 mm/s; resp. 1.8 ± 0.6 mm/s), while arterial BFV reduction was more pronounced in the long-term group (2.5 ± 0.5 mm/s; resp. 3.1 ± 0.9 mm/s). These findings seem to point out that even primarily venous retinal vasculitis involves both vascular systems in the course of the disease; furthermore, while venous vascular changes occur very fast after vasculitis onset, the arteries are also affected, especially in the progressed stages, as already described for BSCR-related retinal vasculitis [3,5,6].

This ischemic effect on the retinal vasculature could also be qualitatively visualized with the RFI when using non-invasive capillary perfusion maps (nCPMs), as already shown with the nCPMs in different stages of diabetic retinopathy [8,9,30,33,35]. The nCPMs in our retinal vasculitis patients revealed a reduced number of retinal arterioles and venules, a lower degree of vessel complexity and branching patterns, as well as an enlarged foveolar avascular zone (Figure 4B,D). In particular, retinal vessel reduction and capillary non-perfusion were even more pronounced in patients with a prolonged inflammatory retinal disease (Figure 4A–D). These alterations are in line with previous data based on FAG and OCT-A [5,6,7,11,13], indicating once again the occlusive effects of retinal vasculitis.

As mentioned above, most RFI studies were performed in patients with diabetic retinopathy (DR). Patients with non-proliferative DR (NPDR) were reported to have decreased BFV in both arterioles and venules compared to the controls. These alterations may be attributed to swelling, distorting, and breaking down of retinal vessels that may occur in the non-proliferative stage of diabetic retinopathy [9,36,38]. Although the patients with proliferative diabetic retinopathy (PDR) also disclosed reduced venular BFV, they had an increased arteriolar BFV [23,34]. This was understood as secondary to the neovascular anastomotic shunts traversing capillary closure areas and deviating blood directly from the arterioles to the venules [23,34]. In contrast, none of our RV patients disclosed retinal or optic disc neovascularizations when FA was being performed. This is in accordance with Testi et al. [6], who already stated that the presence of ischemia in BSCR seems not to progress to neovascular complications and may explain the arteriolar BFV reduction found in our RV patients.

Similar alterations were measured with the RFI in some other diseases associated with primary or secondary retinal vasculature inflammation, e.g., Beutelsbacher et al. [55] examined patients with retinitis pigmentosa, Jittpoonkuson et al. [56] had one subject with central retinal vein occlusion (CRVO), Feng et al. [51] studied patients with anterior uveitis, and Jiang et al. [57] analyzed multiple sclerosis (MS) patients. All these studies showed a reduction in arteriolar and venular retinal BFVs. Unfortunately, Jiang et al. [57] did not provide information on whether their multiple sclerosis patients had intermediate uveitis associated with venous retinal vasculitis, which typically occurs in around 10% of MS patients [58].

Altogether, our study has indicated that the Retinal Function Imager may be useful for a quantitative assessment of retinal BFV, especially in patients with retinal vasculitis. In contrast to other imaging modalities, the RFI provides superior retinal BFV measurements on the microvasculature level, which can be important in understanding retinal vasculitis, when in conjunction with precise blood vessel dynamics information.

Limitations of this pilot study include a spectrum of retinal changes with a heterogeneity regarding vascular dynamics and dysregulation due to capillary closure, hypoxia levels, blood viscosity, and disease duration. To better understand the observed alterations, larger studies with more patients and homogenous subgroups appear crucial. Furthermore, it may be of interest to examine the disease course in RV patients prior to and during anti-inflammatory treatment as well as perform RFI imaging in subjects with associated systemic vasculitides, such as Behcet’s syndrome or systemic Lupus erythematodes.

## 5. Conclusions

This study used the RFI—for the first time, to our knowledge—to evaluate BFVs in patients with retinal vasculitis, demonstrating a reduction in venular and arteriolar BFVs compared to controls. These BFV alterations were already achieved in early disease stages and became more pronounced in progressed disease. This study suggests that imaging the retinal vasculature with the RFI may offer a diagnostic and quantifying tool for retinal vasculitis, as well as a potential imaging modality for assessing the disease course and related treatment approaches targeting several retinal vascular diseases.

## Figures and Tables

**Table 2:** Retinal Blood Flow Velocities.

Group	Arterioles BFV (mm/s) (Mean ± SD)	*p*-Value	Venules BFV (mm/s) (Mean ± SD)	*p*-Value	BCVA logMAR (Median ± SD)
Patient group	2.75 ± 0.74	*p* < 0.001 *	1.75 ± 0.51	*p* = 0.016 *	0.1 ± 0.36
Control group	3.79 ± 0.50		2.35 ± 0.44		0 ± 0.01

Long-term uveitis group	2.53 ± 0.48	*p* = 0.416 *	1.69 ± 0.41	*p* = 0.859 *	0.1 ± 0.37
Short-term uveitis group	3.06 ± 0.95		1.83 ± 0.64		0.05 ± 0.15

Long-term uveitis group	2.53 ± 0.48	*p* < 0.001 *	1.69 ± 0.41	*p* < 0.027 *	0.1 ± 0.37
Short-term uveitis group	3.06 ± 0.95	*p* = 0.182 *	1.83 ± 0.64	*p* = 0.395 *	0.05 ± 0.15
Control group	3.79 ± 0.50		2.34 ± 0.43		0 ± 0.01

BFV = Blood Flow Velocity; SD = Standard deviation; BCVA = best corrected visual acuity; logMAR = Logarithm of the Minimum Angle of Resolution. *p*-values adjusted with Tukey´s method for multiple comparisons. * Fitted using mixed regression method.

## Data Availability

Data are available on reasonable request from the corresponding author.
